# Canadian academics’ use of predatory journals

**DOI:** 10.29173/jchla29579

**Published:** 2021-12-01

**Authors:** Maureen Babb

**Affiliations:** Science Liaison Librarian, Sciences and Technology Library, University of Manitoba, Winnipeg, MB

## Abstract

**Introduction:**

Predatory journals have been acknowledged as an increasing concern in the scholarly literature over the last decade, but research on the subject has been sparse. Research that has focused on predatory journals in the Canadian context has been even rarer, and limited to work focused on a single university. This study explores publishing trends in predatory journals by authors affiliated with Canadian universities.

**Methods:**

Articles published by authors at 30 Canadian universities, including all universities in the U15, were pulled from select predatory journals. Key data including author affiliation, article type, discipline, and grant information were extracted from the articles.

**Results:**

All universities in the study were found to have publications in predatory journals. The health sciences accounted for 72% of the publications, and the sciences for 20%. Research articles accounted for 50% of the articles. Opinion, editorial, or commentary pieces accounted for 24% and 19% were review articles. Grant funding was indicated in 34% of the articles, with NSERC and CIHR being top funders. The research-intensive U15 universities were found to publish more in predatory journals than their non-U15 compatriots, even when the universities were of similar size.

**Discussion:**

Canadian scholars were found to publish in predatory journals, particularly those scholars from the health sciences and research-intensive U15 universities. Grant funding was common, and often came from high profile funders like NSERC and CIHR. This study suggests that policy and education initiatives may be warranted in Canadian contexts, especially in the health sciences and at research-intensive universities.

## Introduction

Predatory journals, sometimes called deceptive journals, pseudo-journals, or bad faith journals [1-3], have become a growing concern in the academic publishing landscape since they were brought to the attention of the scholarly community by librarian Jeffrey Beall in 2010 [4, 5]. Despite the fact that concerns about predatory journals had been increasingly raised over the last decade, there was no standardized definition of predatory journals or predatory publishers until late 2019, when a consensus definition was published in *Nature*, identifying them as *“*…entities that prioritize self-interest at the expense of scholarship and are characterized by false or misleading information, deviation from best editorial and publication practices, a lack of transparency, and/or the use of aggressive and indiscriminate solicitation practices.” [6] Even still, this definition has drawn criticism, and is not accepted by all scholars [7].

Over the years since the introduction of the term, many editorials, articles, commentaries, and online posts have been published, often containing dire warnings or heated opinions about predatory journals or publishers [7-9]. Many pieces have provided introductions to predatory journals, and advice on how to spot or avoid publishing in them [10-12]. Others have focused on the degree to which predatory journals have the ability to undermine the scholarly record, academics, institutions, and their ability to influence patient care in clinical settings [13-19]. On the opposite end of the spectrum, some commentators have suggested that concerns over predatory journals are over-blown, are an attempt to discredit the open-access (OA) movement altogether, or that discussion of predatory journals draws attention away from systemic issues in traditional scholarly publishing [20-22]. Other commentators have noted that the language and tools used to discuss and assess predatory journals is racially biased, and discriminates against newer journals, journals from developing countries, and journals from non-English-speaking countries [23, 24]. These last concerns are certainly justified, given commentaries on predatory journals which have suggested that they are only a problem for newer scholars and scholars from less developed nations – a perception that has persisted, despite evidence that scholars of all levels across the globe are impacted [13]. However, despite the polarized views presented in many of the editorials on predatory journals, there has been little empirical research conducted that informs those views. The limited empirical research that has been conducted has mostly occurred since 2017 [25]. It is therefore safe to say that this field of study is still in its infancy, despite the volume of editorial content that has been published on it.

The research to date indicates that large quantities of predatory journals exist, and that their numbers are continuing to expand [5, 13, 26-28]. It also indicates that the reach of these journals is indeed global, and not limited to inexperienced scholars, nor to scholars from developing countries, as had often been assumed previously [13, 15]. Much of this research has used the now-defunct Beall’s list as a mechanism for identifying the predatory journals that their studies were based on [26-36]. As Beall’s list has been heavily criticized for a lack of transparency, and for likely including legitimate journals in its ranks, its use in these studies has been questioned as well; in the view of some, the use of Beall’s list to identify predatory journals negates the findings of any study that used it, as it demonstrates a flawed methodology [37-39]. Despite being shuttered in early 2017, Beall’s list, now hosted on several mirrors across the internet, remains a common tool used to identify predatory journals and publishers [21, 24, 40]. Beall’s list had been used in these studies because identifying predatory journals is difficult and time-consuming, not least because predatory journals exist on a spectrum, and there is no single collection of factors that will identify a predatory journal without fail. For much of the last decade, Beall’s list was also the only major curated list of predatory journals – other lists, such as Cabell’s (paywalled) and Kscien’s now exist, but none have yet reached the level of familiarity or notoriety that Beall’s list possessed [41, 42]. It is reasonable to suggest that the limited number of studies conducted on predatory journals has been in part because of the difficulty of defining predatory journals as a concept, and more specifically, because of the difficulties associated with identifying specific predatory journals to study.

One reason given for the variety of alternative terms for predatory journals mentioned earlier is that ‘predatory’ may be an inappropriate term to describe them, as it is not always the case that authors are ‘preyed on’. Rather, it has been suggested, some scholars may be publishing in them with full knowledge of their nature, in order to pad their CV with legitimate-looking publications, or in order to rush a publication through before a critical grant or promotion deadline [2, 3, 43]. Stemming from this suspicion arises discussion about the pressure to publish, and on the value placed on quantity of publications over quality in the modern academic landscape. There has been speculation that this pressure to publish can be directly linked to the rise and proliferation of predatory journals [30, 32, 43-45]. This study addresses this possibility by exploring the differences in predatory journal publications between Canada’s research-intensive U15 universities and other, non-U15 universities.

The study presented here adds to the growing body of research exploring predatory journals. Several previous studies about predatory journals have been regionally-bound, but only three have focused on a Canadian context – one article explored the use of predatory journal articles in undergraduate student paper bibliographies at the University of Brandon [21], the second explored how faculty at a small business school in Canada used publications in predatory journals to obtain rewards in a professional context [35], and the third was a direct and critical response to the second [46]. No studies of predatory journals have yet focused on a more broadly Canadian context. This study explores the patterns of publication in predatory journals by Canadian scholars. Focusing specifically on publications in OMICS journals, this study addresses the following research questions:

1. What are the patterns of Canadian publications in predatory journals?
  - What trends of publication exist over time?
  - Are there variations in publication habits based on universities?
  - Were the studies published in these journals grant funded?
2. What content do Canadian scholars publish in predatory journals?
  - Are there variations in publication habits based on discipline?
  - What type of content is published? (e.g.; research articles, editorials)
3. Does the research intensity of a university affect the degree to which Canadian scholars publish in predatory journals?
  - Is there a difference between the publication trends of U15 and non-U15 universities?

## Methods

This study looked at publications in OMICS journals from a set of Canadian universities. OMICS journals have been selected to counter the difficulty and subjectivity of reliably identifying predatory journals or publishers. In 2016, the Federal Trade Commission (FTC) in the United States sued OMICS for deceptive practices [47]. The FTC won their case, and OMICS was ordered to pay 51 million dollars, and to change its practices in the future [48]. OMICS appealed this, but the ruling was upheld in 2021. OMICS, a company based in India and not the United States where the FTC is located, has made no indication that it will adhere to the verdict, pay the fine, or change its business practices. Therefore, unlike the grey area surrounding Beall’s list, OMICS may be reasonably identified as a predatory publisher, despite the publisher’s claims to the contrary. In order to avoid the pitfalls associated with relying on Beall’s list to identify predatory publishers, this study has chosen to limit the journals in its sample to those published by OMICS. OMICS publishes a wide array of journals in a variety of subject areas and was founded in 2007.

Thirty Canadian universities from which to examine publications were selected, including all of the U15 universities. The U15 universities were selected as they represent Canada’s most research-intensive universities, similar to the R1 University category in the United States. As such, these institutions place a high value on producing research and scholarship, and on obtaining grant funding. Because it has been theorized that publication in predatory journals is linked to the pressure to publish, including research-focused institutions was critical. These U15 universities required a point of comparison, and so fifteen non-U15 universities were selected for inclusion as well. The other fifteen universities were selected by ‘matching’ them to the U15 universities – for every U15 university included in the study, a non-U15 university from the same province was selected for inclusion. The universities included in this study are outlined in table 1.

**Table 1:** – Sampled Universities.

U15 Universities	Student Population	Non-U15 Universities	Student Population
University of Alberta	39,502	Athabasca University	39,700
University of British Columbia	50,330	Simon Fraser University	35,204
University of Calgary	29,860	Mount Royal University	24,768
Dalhousie University	18,354	Acadia University	4,254
Université Laval	37,800	Université du Québec à Montréal	41,670
University of Manitoba	26,800	University of Winnipeg	9,847
McGill University	32,514	Université de Sherbrooke	19,500
McMaster University	26,070	Wilfrid Laurier University	20,000
Université de Montréal	55,540	Concordia University	43,944
University of Ottawa	42,587	Ryerson University	38,560
Queen’s University	20,550	Brock University	17,006
University of Saskatchewan	18,620	University of Regina	12,170
University of Toronto	74,760	York University	52,290
University of Waterloo	35,100	Carleton University	24,250
Western University	34,100	University of Guelph	22,080

It should be noted that the purpose of ‘matching’ the universities in this way was simply a means of selecting 15 non-U15 universities to include in the study in order to create a pool of non-U15 universities that, as a unit, are similar to the U15 pool. No attempt was made to ensure that a given university was similar to its ‘match’ – not in terms of university culture, language, or indeed, size. Though the universities were matched as closely as possible based on size, this still led to massive discrepancies in sizes between some U15 universities and their ‘match.’ Overall, however, the two pools show considerable overlap in terms of student and graduate student populations – though the high end of scale is dominated by the U15 and the low end by the non-U15 universities. Universities were never compared directly to their ‘match’ at a one-to-one level. Comparisons were only made at a group level; U15 vs. non-U15.

All findable articles published by authors affiliated with the aforementioned universities published in OMICS journals were collected. The OMICS website (https://www.omicsonline.org/ ) includes an internal search function that allows users to search through their publications for specific content. This internal search function was used to identify the articles collected for this study. For each university included, a list of ways that the university name may have been written in an author affiliation section was compiled – this included English and French versions of names of French-speaking universities (e.g.; ‘University of Montreal’ and ‘Université de Montréal’), versions of university names with and without relevant accents (e.g.; ‘Université de Montréal’ and ‘Universite de Montreal’), and current and previous versions of university names where applicable (e.g.; ‘University of Western Ontario’ and ‘Western University’). Potential versions of affiliations for which it would not have been possible to verify which university it was referring to were not included (e.g.; ‘U of A’). Each of the identified terms were entered individually into the OMICS internal search, and PDF versions of every article returned for each search were downloaded. Results were not limited to certain years, and included all results from the earliest OMICS publications to the date of data collection; 2008-2019. Data collection occurred between May 2^nd^ and June 12^th^, 2019. Data extraction from the articles began once they had been downloaded, and included:

- Content Type (e.g.; Research article, editorial)
- Title of Article
- Authors
- Author Affiliations
- Name of Journal
- Year of Publication
- Discipline (e.g.; Nursing)
- Broad Discipline (e.g.; Health Sciences)
- Grant Statement

Articles were excluded if they did not include at least one author affiliated with any of the thirty sampled universities.

Once data extraction was complete, results were analyzed using both descriptive statistics and exploratory data analysis. Because this study looked at the entirety of OMICS collections and was not limited to any specific discipline, no distinction was made regarding author order, as this varies by discipline; some disciplines place the primary author first, others last, and some disciplines place authors in alphabetical order. All authors were therefore considered equally important, as it was not always possible to tell who the primary author of any given article was.

Data has been anonymized for the write up of this study; identifying details of the articles and their authors have not been included – publication in predatory journals such as OMICS journals has potential to negatively impact a scholar’s career, if those publications become known. It is not the intention of this study to directly impact anyone’s career in such a way, and as such, that information will not be provided.

## Results

**Fig. 1:**
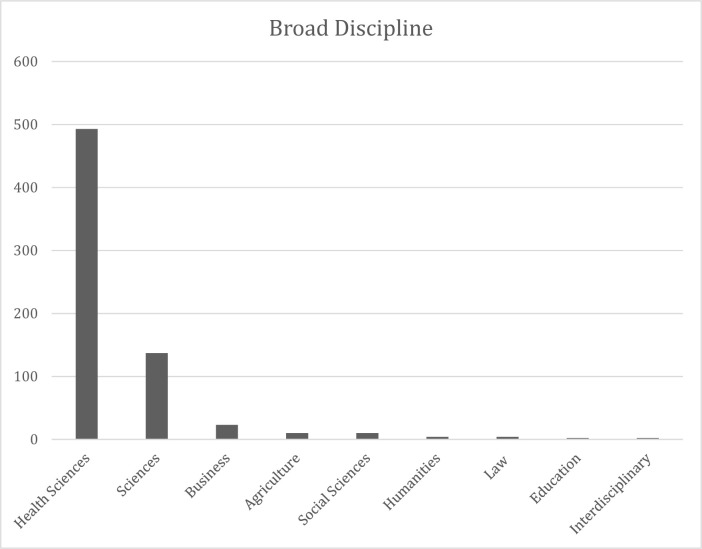
– Broad Discipline

A total of 686 articles, featuring 1457 distinct authors from the 30 sampled universities were identified. Many of the articles also had authors not affiliated with the 30 sampled universities; however, these authors were not included in the results. The bulk of these articles (n = 493, 72%) were published in health sciences disciplines, followed distantly by sciences (n = 137, 20%), and business (n = 23, 3%). Social sciences, agriculture, law, humanities, education and interdisciplinary areas each accounted for 10 or fewer articles. These results are presented in figure 1.

**Fig. 2:**
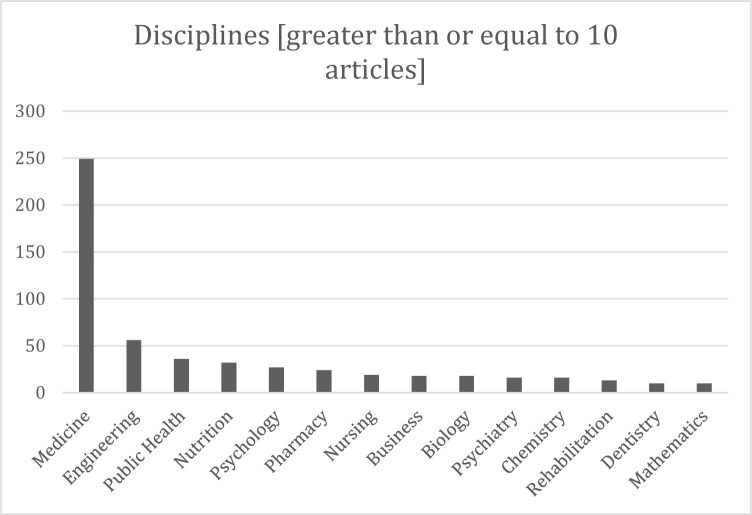
– Disciplines.

In terms of specific fields, the most sizable number of articles were in medicine (n = 249, 36%). The field with the next highest number of publications was engineering, with 56 (8%) articles. Most fields identified in the data had fewer than 10 publications. Specific fields with ten or more articles are presented in figure 2.

The articles published represented a number of categories, including research article, review article, and editorial/commentary/opinion. Research articles represent exactly half (n = 343, 50%) of the total number of articles. The other half of the publications are divided across different content types, detailed in table 2. Editorial/commentary/opinion pieces and review articles, such as systematic or scoping reviews, also make up significant portions of the results.

**Table 2:** – Types of Publications.

Type of Publication	Number	Percentage
Research Article	343	50%
Editorial/Commentary/Opinion	164	24%
Review Article	131	19%
Case Report	38	6%
Letter to the Editor	6	1%
Medical Image	2	0.2%
Book Review	1	0.1%
Protocol	1	0.1

Just over one third of the articles (n = 231, 34%) reported receiving grant funding for their project; several articles reported receiving funding from more than one source. The greatest percentage (n = 111, 48%) of these grants were obtained from an array of non-corporate granting agencies, including local governments or non-profit organizations in Canada, but excluding Tri-council and NRC-CNRC (National Research Council of Canada) grants, as these are major granting agencies in Canada and were tracked separately. NSERC (National Sciences and Engineering Research Council of Canada) and CIHR (Canadian Institutes of Health Research) respectively provided the next highest number of grants to support the work included in these articles at 75 (33%) and 70 (30%), respectively. Many articles included funding from multiple sources. The breakdown of granting agencies is included in table 3.

**Table 3:** – Granting Agencies.

Granting Agency	Number	Percentage
Non-Corporate Grant	111	48%
NSERC	75	33%
CIHR	70	30%
Grant from another Country	34	15%
Internal University Grant	31	13%
Corporate Grant	27	12%
NRC-CNRC	4	2%
SSHRC	3	1%
Grant from an International Body	3	1%

**Fig. 3:**
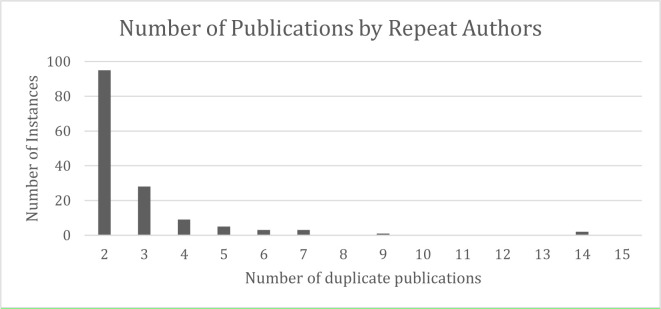
– Repeat Authors

The majority of authors from the 30 surveyed institutions did not publish in OMICS journals more than once, however 136 authors (9%) were repeat authors. While more repeat authors came from the U15 in terms of sheer numbers (n = 119, 88%), the percentage of repeat authors did not vary between U15 and non-U15 universities; in both cases, repeat authors represented 9% of the total number of authors. Most repeat authors appeared only twice – that is, they had published only two times in OMICS journals. The number of times that repeat authors published in OMICS journals may be observed in figure 3. The most publications in OMICS journals from a repeat author was 14, a number which two separate authors obtained.

**Fig. 4:**
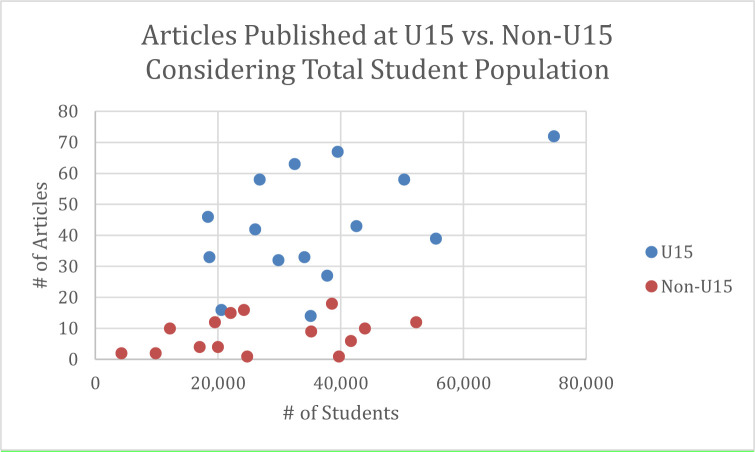
– Publications at U15 and Non-U15 Universities Considering Student Population.

**Fig. 5:**
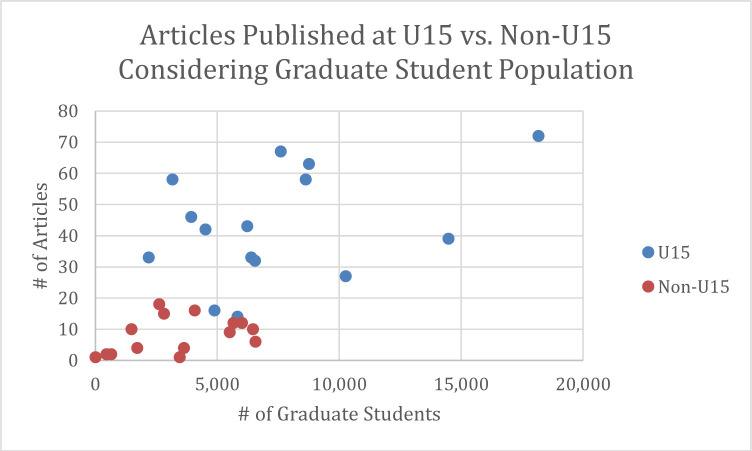
– Publications at U15 and Non-U15 Universities Considering Graduate Student Population.

U15 universities published considerably more articles than non-U15 universities. This pattern held true even when universities were of comparable size according to total number of students (figure 4) or graduate students (figure 5). Given the prevalence of articles published in the health sciences, the presence of a faculty of one or more of the health sciences (e.g.; Medicine, Dentistry, Health Sciences, Nursing) at the universities was considered as a possible confounding factor; however, the presence or absence of a faculty relating to the health sciences did not appear to affect the results.

**Fig. 6:**
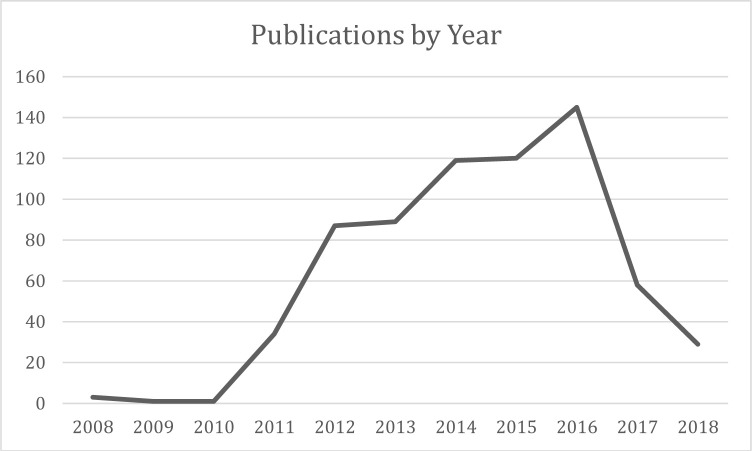
– Publications over time.

The earliest publications from the sampled universities appeared in 2008, one year after the founding of OMICS. In the early years, publications from the sampled universities were sparse, but began climbing in 2011, when the number of publications from the sampled universities jumped from only one in the previous year to thirty-four. From there, publications continued to rise steadily, until they reached a peak in 2016, with 145 publications from sampled universities. After this, there is a steep decline in publications from sampled universities, dropping to 29 in 2018. No publications were identified from 2019, despite data collection occurring mid-way through the year (figure 6). It is worth noting here that this sudden decline is unlikely to represent a general trend across publications in predatory publishers broadly speaking – 2016 was the year that the FTC charged OMICS publishers with deceptive practices, an event that was widely publicized in academic circles. The post-2016 decline is therefore likely to be limited to the selected sample.

## Limitations

There are a number of limitations within this study. One of these relates to the use, during data collection, of the OMICS internal search engine. The precise quality of the search engine is not clear, but it is obvious that the search is prone to errors of omission; articles that by rights should have appeared when using the search terms for one sampled university did not, and are included in this study only because they appeared in the search for articles from another sampled university. It is unclear how many of these errors of omission occurred that were not caught by luck in another search conducted for the study. It is likely, then, that the true number of articles published by the sampled universities is higher than the number reported in this study. The OMICS internal search often also returned articles that had no apparent reason to be included in the search results, suggesting that the search engine is prone to additional error types. The search was consistent over time, however, returning the same results for the same searches.

This study considers only the content of one publisher. Though this choice was a considered one, it nonetheless means that the results of this study have limited ability to be generalized to represent publications in predatory journals more broadly. Likewise, because only one publisher is included, there can be no sense of scope of Canadian publications in predatory journals – it is obvious from the results that all sampled universities have publications in predatory journals, but it cannot provide a suggestion of the extent to which that occurs. Related to this is the fact that there is no way to know what percentage of the sampled universities’ scholarly output is represented in this study, and no way to know how that percentage would change with the consideration of other predatory publishers. Because of this, it is also impossible to know whether the results indicate that U15 universities are using predatory journals at a higher *rate*, or if the higher number of publications in predatory journals merely reflects a higher number of publications from U15 universities in general.

The non-U15 universities were selected according to student size and province-matching. It is possible that another mechanism for selecting the pool of non-U15 universities may have been more ideal; for example, attention could have been paid to university language, culture, remoteness, mechanisms of course delivery, age of university, or core subject offerings. It is possible that using a different pool of non-U15 universities, or including all non-U15 universities in Canada in the study would have yielded different results.

It is also unclear what affiliation authors have with the university associated with their name. While in some cases, authors listed their department and/or status (e.g.; Assistant Professor), others merely included the name of the university. Without extensive and time-consuming follow-up, there is no way to know whether the authors were faculty, graduate students, something else, or indeed, whether they actually were affiliated with the university in question at all.

## Discussion and Conclusions

There has been a popular perception in discussions surrounding predatory journals that developed, ‘Western’ countries such as Canada need not concern themselves as those that publish in predatory journals generally come from developing nations [13, 15]. While there is some evidence that predatory publication rates are higher in developing countries, previous studies have shown that those from developed, ‘Western’ countries do publish in predatory journals – something this study confirms [15, 27, 28, 49-51]. While this study cannot demonstrate the extent, it is clear that publication in predatory journals happens in Canada; every university in this study had publications in the target journals. Beyond this, the results of this study suggest that a significant portion of projects published in predatory journals were grant funded; over one third of the papers analyzed in this study were supported by grants, frequently from major national funders. As has been pointed out by other authors, the presence of grant-funded studies in predatory journals is particularly concerning, as it represents a waste of financial resources, often public resources, in addition to researcher time and effort to publish in journals that do not meet the standards of proper scholarly publishing [14, 15].

The vast majority of the publications in this sample came from the health sciences, with the sciences a distant second, and other subject areas being negligible in comparison. This parallels the number of journals that OMICS publishes in each category; 68% of their journals are published in the health sciences and 28% in the sciences. It is unclear from this study whether the focus on the health sciences and sciences is unique to the OMICS publisher, or if it would be found across a broader sample of predatory journals. The reason OMICS publishes more health sciences and sciences is unknown, though educated speculations may be made; namely, the health sciences, and to a lesser degree the sciences, are comparatively well funded, and therefore, may be more targeted by predatory publishers. This speculation cannot be confirmed without further study, however. That health sciences research appears to be a target for predatory publishers should be cause for concern, as ensuring both the quality and adequate distribution and preservation of health sciences research can have direct impacts on patient care and human life.

It also appears that authors at U15 universities are more at risk of publishing in predatory journals than their non-U15 counterparts, regardless of university size. As with the higher occurrence of publications in the health sciences, it is unclear why this is the case – it may simply relate to a higher overall research output at U15 universities, or it may represent something more sinister, such as a willingness of more faculty to publish in dubious journals to survive in a publish-or-perish environment. Determining the reason for this difference between U15 and non-U15 institutions will require further research.

Repeat authors account for only 9% of the total number of authors, and of those, the vast majority only had two OMICS publications to their name. This may indicate that the majority of authors are not using publications in these journals for nefarious purposes, but rather, that they submitted without realizing the nature of the publisher. This cannot be confirmed without further exploration, but previous studies on author motivation for publishing in predatory journals suggest that lack of awareness about predatory journals is a common experience of authors who submit to them [30, 32, 40]. In previous studies, some authors did use predatory journal publications to game the system [32, 43], and the fact that a handful of authors published up to 14 times in OMICs journals suggests that these motives may also be at work in this sample as well.

The possibility that a lack of awareness about predatory journals may have been the case for the majority of the authors in this sample is perhaps strengthened by the sharp drop-off in publications in the sample after 2016, when OMICS was very publicly sued by the FTC; once enlightened to the fraudulent practices of the publisher, fewer sought to publish with them. This additionally suggests that awareness-raising efforts regarding predatory journals may be an effective tool to combat them – previous work has suggested that awareness levels about predatory journals are low in academia and in the health sciences, and that education on the subject can raise awareness [52-55].

This exploratory study raises questions that cannot be answered with the data available, including; are the health sciences truly as disproportionately affected by predatory journals, as this study suggests? If so, why? What reasons exist to explain why scholars at the U15 appear to publish more frequently in predatory journals? Does raising awareness of predatory journals among scholars prevent publication in predatory journals, and if so, what methods of awareness raising are most effective? Such questions are potential areas of future research.

This study also raises questions that cannot be answered by further research, but which must be addressed by policy-makers instead – this study suggests that high percentages of papers published in predatory journals represent studies that have obtained grant funding. What implications does this have for granting agencies going forward? Additionally, while this study only looked at a single publisher, every university in the sample was represented in the data – what implications does this have for universities across Canada? What implications does it have for measures of research assessment used in Canadian universities and cultures of publish or perish?

This study has provided a first look into patterns of use of predatory journals by Canadian scholars at U15 and non-U15 universities. It has highlighted disturbing trends that warrant further investigation, notably high usage of predatory journals by scholars in the health sciences and at U15 universities. It has raised questions to be addressed in future studies and by policy-makers. Predatory journals remain an issue in scholarly publishing that is heavily discussed, but scarcely researched. This paper adds insight to the Canadian dimension of predatory publishing.
